# Supplementary material for: Cost-Effective Protein Production in CHO Cells Following Polyethylenimine-Mediated Gene Delivery Showcased by the Production and Crystallization of Antibody Fabs
Source: Antibodies (Basel). 2023 Aug 4;12(3):51. doi: 10.3390/antib12030051 (PMC10443350; doi:10.3390/antib12030051)
Supplement: Supplementary file 1 [file antibodies-12-00051-s001.zip › Supplementary Table S1.pdf]

Supplementary Table S1 pCMV-3UTR cloning site enzymes and alternative cloning possibilities

pCMV-3UTR cloning site enzyme	Isoschizomers	Compatible sticky ends*	Recognition sequences
AgeI	AsiGI, BshTI,	AccIII, Aor13HI, BseAI, Bsp13I, BspEI, Kpn2I, MroI	5′…T●CCGG A…3′<br>3′…A GGCC●T…5′
5′…A●CCGG T…3′<br>3′…T GGCC●A…5′	CspAI, PinAI	Cfr9I, TspMI, XmaI, XmaCI	5′…C●CCGG G…3′<br>3′…G GGCC●C…5′
		MroNI, NgoMIV	5′…G●CCGG C…3′<br>3′…C GGCC●G…5′
		BsaWI	5′…W●CCGG W…3′<br>3′…W GGCC●W…5′
		Bse118I, BsrFI, BssAI, Cfr10I	5′…R●CCGG Y…3′<br>3′…Y GGCC●R…5′
		MreI	5′…CG●CCGG CG…3′<br>3′…GC GGCC●GC…5′
		SgrAI	5′…CR●CCGG YG…3′<br>3′…GY GGCC●RC…5′
SpeI	AhlI, BcuI	AspA2I, AvrII, BlnI, XmaJI	5′…C●CTAG G…3′<br>3′…G GATC●C…5′
5′…A●CTAG T…3′<br>3′…T GATC●A…5′		AsuNHI, NheI	5′…G●CTAG C…3′<br>3′…C GATC●G…5′
		XbaI	5′…T●CTAG A…3′<br>3′…A GATC●T…5′
EcoRI		MluCI, Sse9I, TasI, Tsp509I, TspEI	5′…●AATT …3′<br>3′… TTAA●…5′
5′…G●AATT C…3′<br>3′…C TTAA●G…5′		MfeI, MunI	5′…C●AATT G…3′<br>3′…G TTAA●C…5′
		AcsI, ApoI, XapI	5′…R●AATT Y…3′<br>3′…Y TTAA●R…5′
XhoI	PaeR7I,	Ama87I, AvaI, BmeT110I, BsiHKCI, BsoBI, Eco88I, SmlI, SmoI	5′…C●TCGA G…3′<br>3′…G AGCT●C…5′
5′…C●TCGA G…3′<br>3′…G AGCT●C…5′	Sfr274I, SlaI	SalI	5′…G●TCGA C…3′<br>3′…C AGCT●G…5′
		SgrDI	5′…CG●TCGA CG…3′<br>3′…GC AGCT●GC…5′
		AbsI	5′…CC●TCGA GG…3′<br>3′…GG AGCT●CC…5′
		PspXI	5′…VC●TCGA GB…3′<br>3′…BG AGCT●CV…5′

* from the SnapGene Viewer v7.0.1 (SnapGene software (www.snapgene.com)) enzyme database

B = C or G or T
R = A or G
V = A or C or G
W = A or T
Y = C or T
