# Supplementary material for: Cost-Effective Protein Production in CHO Cells Following Polyethylenimine-Mediated Gene Delivery Showcased by the Production and Crystallization of Antibody Fabs
Source: Antibodies (Basel). 2023 Aug 4;12(3):51. doi: 10.3390/antib12030051 (PMC10443350; doi:10.3390/antib12030051)
Supplement: Supplementary file 1 [file antibodies-12-00051-s001.zip › Supplementary Table S4.pdf]

Supplementary Table S4 Comparison of cost for ExpiFectamine and PEI gene delivery for TGE in CHO cells

Method of gene delivery	Cost per 25 ml culture (EUR)	Minimal initial investment (EUR)	Transfected volume (l)*	Note
ExpiFectamine CHO Transfection Kit	36	1430	1	ThermoFisher Scientific, USA, Cat. No. A29129
PEI method (this work)	2.5	869	4.4#	
PEI	0.5	209	12.5	Polysciences, USA, Cat. No. 23966-100
feed	2	660	4.4	this work

* calculated for the amount of material corresponding to the minimal investment

# limiting factor is the amount of CHO CD Efficient Feed A (see Materials and Methods)
