# Supplementary material for: Cost-Effective Protein Production in CHO Cells Following Polyethylenimine-Mediated Gene Delivery Showcased by the Production and Crystallization of Antibody Fabs
Source: Antibodies (Basel). 2023 Aug 4;12(3):51. doi: 10.3390/antib12030051 (PMC10443350; doi:10.3390/antib12030051)
Supplement: Supplementary file 1 [file antibodies-12-00051-s001.zip › Supplementary Tables S2 and S3.pdf]

Supplementary Table S2 Crystallization conditions

	DC11Fab	complex I (MN423Fab + dGAE)	complex II (DC11Fab + DC25Fab + dGAE)
Method	Vapor diffusion	Vapor diffusion	Vapor diffusion
Plate type	MRC 2-well	EasyXtal	MRC 2-well
Temperature (K)	295 K	295 K	295 K
Protein concentration	8 mg/ml	4 mg/ml	5 mg/ml
Buffer composition of protein solution	10 mM Tris-HCl, pH 7.2, 50 mM NaCl	10 mM Tris-HCl, pH 7.2, 50 mM NaCl	10 mM Tris-HCl, pH 7.2, 50 mM NaCl
Composition of reservoir solution	0.1 M MIB pH 6.0, 25 % (w/v) PEG 1500	0.1 M MIB pH 8.0, 25 % (w/v) PEG 1500	0.2 M ammonium sulfate, 0.1 M sodium acetate pH 5.6, 25 % (w/v) PEG 4000
Volume and ratio of drop	0.5 µl + 0.5 µl (1:1)	0.6 µl + 0.6 µl (1:1)	0.2 µl + 0.2 µl (1:1)
Volume of reservoir	80 µl	200 µl	80 µl

MIB – malonic acid : imidazole : boric acid in 2:3:3 molar ratio

Supplementary Table S3 Data collection and processing

Values for the outer shell are given in parentheses.

	DC11Fab	complex I (MN423Fab + dGAE)	complex II (DC11Fab + DC25Fab + dGAE)
Diffraction source	EMBL-DESY P13	SLS-PSI PXI	EMBL-DESY P13
Wavelength (Å)	0.9763	0.9999	0.9763
Temperature (K)	100	100	100
Detector	DECTRIS EIGER 16M	DECTRIS EIGER 16M	DECTRIS EIGER 4M
Crystal-detector distance (mm)	160.248	201.035	144.365
Rotation range per image (°)	0.1	0.1	0.1
Total rotation range (°)	360	270	360
Exposure time per image (s)	0.008	0.00125	0.008
Space group	P2 (number 3)	$P2_1$ (number 4)	$P2_12_12_1$ (number 19)
a, *b*, *c* (Å)	40.9, 88.4, 57.4	68.23, 36.55, 84.92	41.75, 94.37, 200.01
α, β, γ (°)	90.0, 94.8, 90.0	90.0, 109.63, 90.0	90.0, 90.0, 90.0
Mosaicity (°)	0.072	0.088	N.A.
Resolution range (Å)	48.06-1.30 (1.38-1.30)	43.47-1.45 (1.54-1.45)	42.67-2.70 (2.83-2.70)
Total No. of reflections	670292 (107088)	353325 (50285)	149653 (20270)
No. of unique reflections	95153 (14882)	69188 (10693)	22626 (2943)
Completeness (%)	95.4 (92.5)	97.9 (94.2)	99.9 (99.9)
Redundancy	7.0 (7.2)	5.1 (4.7)	6.6 (6.9)
$\langle I/\sigma(I)\rangle$	7.7 (0.6)	8.9 (0.6)	9.5 (2.2)
R_{meas}	0.124 (2.845)	0.123 (2.233)	0.160 (0.912)
Overall *B* factor from Wilson plot ($Å^2$)	24.9	15.3	45.6
